# Multimodal Assessment of the Prognostic Role of Ectopic Inner Foveal Layers on Epiretinal Membrane Surgery

**DOI:** 10.3390/jcm12134449

**Published:** 2023-07-02

**Authors:** Carlo Gesualdo, Settimio Rossi, Clemente Maria Iodice, Andrea Rosolia, Paolo Melillo, Michele Della Corte, Francesca Simonelli

**Affiliations:** Eye Clinic, Multidisciplinary Department of Medical, Surgical and Dental Sciences, University of Campania “Luigi Vanvitelli”, Via S. Pansini, 5, 80131 Naples, Italy; carlo.gesualdo@unicampania.it (C.G.); settimio.rossi@unicampania.it (S.R.); dr.rosolia@gmail.com (A.R.); paolo.melillo@unicampania.it (P.M.); michele.dellacorte@unicampania.it (M.D.C.); francesca.simonelli@unicampania.it (F.S.)

**Keywords:** ectopic inner foveal layers, epiretinal membrane, microperimetry, multifocal electroretinogram, multimodal imaging, pars plana vitrectomy, vitreoretinal surgery

## Abstract

Background: To perform a multimodal assessment of the ectopic inner foveal layers’ (EIFL) prognostic role on idiopathic epiretinal membrane (ERM) surgery. Methods: We retrospectively followed-up for 12 months 27 patients who underwent ERM surgery and stratified them based on EIFL presence (group 1) or absence (group 2) at baseline. Central Retinal Thickness (CRT) and best-corrected visual acuity (BCVA) were compared pre- and post-operatively at 1, 4 and 12 months, whereas fixation stability (FS), macular sensitivity (MS) and multifocal electroretinogram (mfERG) responses were confronted at baseline and 12 months. Results: In group 1, BCVA improved at 4 and 12 months (MD = 0.14 (SE = 0.04); MD = 0.13 (SE = 0.05), respectively) as well as in group 2 (MD = 0.31 (SE = 0.07); MD = 0.41 (SE = 0.08), respectively). CRT did not change in group 1, whereas it decreased in group 2 at 4 and 12 months (MD = −73.13; SE = 23.56; MD = −76.20; SE = 23.56). MS showed no changes in both groups after surgery. FS did not change in group 1, whereas group 2 improved FS 2° (+8.91 ± 13.97) and FS 4° (+4.33 ± 3.84). MfERG P1 wave did not change in group 1, while in group 2 αP1-2, αP1-3 and αP1-4 improved postoperatively (27.97 ± 27.62; 12.51 ± 17.36; 10.49 ± 17.19, respectively). Conclusions: Multimodal assessment confirmed that EIFL negatively affected ERM surgery outcomes.

## 1. Background

The epiretinal membrane (ERM) is an avascular pre-retinal layer made up by abnormal fibro-cellular growth at the vitreoretinal interface, with a prevalence that increases with age, ranging from 7 to 28.9% [1,2]. ERM may be asymptomatic, but when it involves the foveal region, it usually causes a spectrum of symptoms ranging from blurred vision and metamorphopsia up to a significant reduction of best-corrected visual acuity (BCVA) [1,2]. 

ERM management is currently limited to either watchful waiting or to the vitreoretinal surgical approach. Traditionally, the decision to aim for surgery rather than observing the patient was based upon the severity of symptoms and the impact of the ERM on visual function. Conversely, the appearance of the retinal profile on optical coherence tomography (OCT) has never been considered [1,2]. 

Indeed, a significant mismatch between the OCT findings and the patients’ visual function has been described in the recent literature, which reports some cases that were found to have excellent vision with no complaints, despite exhibiting an extensive ERM that significantly affected their retinal architecture [3]. In addition, it is noteworthy that ERM is often reported to affect only one eye, thereby strikingly reducing the evidence of symptoms, which may be surprisingly masked by the preserved good function in the fellow eye [3].

Several authors have questioned the traditional practice of opting for surgery only in cases of advanced ERM stages with significantly reduced vision, advocating the necessity of an early approach for this disease [2,3]. Indeed, post-operative visual outcomes were significantly demonstrated to be correlated to the preoperative BCVA, which was inevitably described as sharply deteriorating in cases where the disease was left untreated. From this perspective, overall patients’ recovery potentialities may clearly benefit from an prophylactic surgery rather than a later rescue [3]. 

Currently, there is a substantial lack of standard recognized biomarkers to evaluate the process of choosing whether to opt for prompt surgery or for a watchful wait. Several biomarkers have indeed been proposed to predict the visual and morphological outcomes of ERM surgery, such as ellipsoid zone (EZ) integrity and photoreceptor outer segment length [4,5,6]. However, the prognostic ability of such parameters often results as insufficient, especially considering the relatively high rate of outer retinal artifacts on SD-OCT evaluation [4,5,6].

In the last few years, the focus of the preoperative evaluation has been progressively moved to the analysis of the inner retinal layers, suggesting that the loss of their normal structure may be considered as a negatively prognostic factor prior to surgery [7,8].

With this purpose, Govetto et al. recently described an Optical Coherence Tomography (OCT) based classification that explores in detail the role of the inner retinal appearance in predicting the evolution of the disease and the outcomes of pars plana vitrectomy (PPV) [9]. The continuous traction realized by the ERM was reported to induce a reorganization of the inner retina, leading to the formation of ectopic inner foveal layers (EIFL), an aberrant extension to the entire foveal region of the inner nuclear layer (INL) and inner plexiform layer (IPL) [9]. The proposed staging scheme is divided into four stages: (1) foveal pit presence with well-defined retinal architecture; (2) foveal pit absence with well-defined retinal architecture; (3) EIFL presence, with foveal pit absence and well-defined retinal architecture; (4) EIFL presence, with foveal pit absence and completely disrupted retinal architecture [9].

Some authors have demonstrated that the baseline visual acuity is strictly and negatively related to the EIFL presence, resulting in minimally impaired values for stages 1 and 2 and significant sharp decline in both stages 3 and 4, which are indeed characterized by the EIFL appearance [9,10]. Moreover, surgical results after a 12-month follow-up reported that both the presence and thickness of EIFL are also negatively correlated with the postoperative BCVA and central foveal thickness (CFT) values [11,12]. In addition, micro-perimetric results showed that a significant post-surgical mean retinal sensitivity (MRS) improvement occurred only in eyes with no described EIFL presence (Stages 1 and 2) [12]. 

With regards to multifocal electroretinography (mfERG), which detects and measures the central retinal electrical activity responses, the results reported in the literature after ERM surgery are controversial, and none investigated the actual influence of EIFL presence on mfERG results. 

Therefore, the present study aimed to evaluate how the EIFL would affect both the functional and morphological outcomes of idiopathic ERM surgery with a multimodal approach [13,14]. Additionally, EIFL influence on mfERG responses and fixation stability (FS) was assessed, for the first time to our knowledge, both pre- and post-operatively [13,14].

## 2. Materials and Methods

A retrospective, observational consecutive chart review of 27 eyes of 27 patients diagnosed with idiopathic ERM was conducted. Patients were visited between January 2020 and January 2022 by two retina specialists (S.R. and C.G.) in the Medical Retina Unit of University of Campania “Luigi Vanvitelli” and all the examinations were retrospectively evaluated by two independent expert observers (C.G. and C.M.I.), requiring a third independent assessment in case of contrasting opinions.

The study was approved by the local ethics committee and performed in compliance with the declaration of Helsinki and international guidelines. 

Each patient was thoroughly informed of surgery risks and benefits and a written informed consent was acquired.

The same experienced vitreoretinal surgeon (M.D.C.) performed all the surgeries, consisting of a 27-gauge pars plana vitrectomy with triamcinolone assisted ERM removal and ILM peeling. Phakic patients underwent vitrectomy with concomitant cataract removal and implantation of an intraocular lens (IOL), so that the final visual outcome would not be affected by cataract formation. 

Patients presenting with any of the following criteria were excluded: refractive error ≥ ±6.0 diopters; history of trauma or ocular surgery; highly graded cataract; glaucoma and any other optic neuropathy; secondary ERM; choroidal neovascularization of any etiology; diabetic retinopathy; retinal vascular occlusion; central serous chorioretinopathy (CSCR); macular hole; macular telangiectasia; age-related macular degeneration (AMD). 

Information including age, gender, and lens status (phakic, pseudo-phakic or aphakic) was collected at baseline for each patient enrolled (Table 1). 

All the patients underwent four examinations (baseline, 1, 4 and 12 months after surgery) that included a check on any previous clinical documentation and a complete eye visit with BCVA evaluation, anterior segment slit lamp bio-microscopy, binocular indirect ophthalmoscopy, and Spectral Domain Optical Coherence Tomography (SD-OCT). Microperimetry (MP-1) and Multifocal Electroretinogram (mfERG) were performed only at baseline and at 12-month postoperative follow-up.

OCT was carried out using Cirrus HD-OCT (Carl Zeiss, Dublin, CA), setting an acquisition protocol that consisted of a five-line raster scan and a macular cube scan pattern (512 × 128 pixels) in which a 6 × 6-mm region of the central retina was scanned within a timeframe of 2.4 s. In all patients, both the central retinal thickness (CRT) and the presence of ectopic inner foveal layers (EIFL) were recorded. CRT was assessed by measuring the distance between the vitreoretinal surface and the retinal pigment epithelium (RPE) at the foveal center. EIFL OCT-based definition consisted of the presence of a continuous hypo/hyperreflective band extending from the inner nuclear layer (INL) and inner plexiform layer (IPL) across the fovea [9]. Patients were stratified into 4 different stages, according to the classification proposed by Govetto et al. [9]. Individuals showing stages 3 and 4 EIFL were combined into the group 1 (EIFL presence), whereas stages 1 and 2 EIFL were included into group 2 (No EIFL).

A MP-1 micro-perimeter (Nidek Technologies, Italy) was used to assess the mean sensitivity (MS) of the 12° central macular area and the percentage of fixation points falling within the 4 and the 2 central degrees circles (FS 2° and FS 4°). 

MfERG was performed using the VERIS technique (Version 3, EDI, Redwood City CA, USA) according to the published guidelines of the International Society for Clinical Electrophysiology of Vision [15]. The P1 waves amplitude and latency within the first four rings were evaluated (αP1-1; αP1-2; αP1-3; αP1-4).

## 3. Surgical Procedure 

Before starting each surgery, the eyelid, the periorbital skin, and the ocular surface were sterilized with a solution of 5% povidone iodine. Patients were administered with retro bulbar block (1% lignocaine and 0.5% bupivacaine) and, in some exceptional cases, general anesthesia as per requirement. Bupivacaine 0.5% was given as a sub-tenon injection in addition to the local anesthesia when needed. 

Pars plana vitrectomy (PPV) was performed within 4 ± 1 weeks from the baseline visit and all the surgeries were carried out by the same experienced vitreoretinal surgeon (M.D.C). A non-contact wide angle viewing system (BIOM, Oculus Inc., Wetzlar, Germany) was adopted for the visualization of the posterior segment. All the patients displaying significant cataracts underwent routine phacoemulsification followed by intraocular lens implantation via 2.2 mm clear corneal incisions. All cataract surgeries were completed before inserting the trocars. 

A 3-port 27-gauge PPV with ERM and ILM peeling was performed with the Constellation 27 G+ Total Plus Vitrectomy System (Alcon Laboratories, Fort Worth, TX, USA). After slightly displacing the conjunctiva, trocars were inserted through both the conjunctiva and the sclera at 3.5 mm from the limbus. Sclerotomies were performed using the trocar cannula with a biplanar entry, first piercing the tissue tangentially and then perpendicularly to the plane in order to create a self-sealing incision, as much as possible. 

Surgery started with a core vitrectomy. Triamcinolone acetonide was injected to facilitate the visualization of the vitreous base, which was meticulously shaved with the help of a scleral depressor. Membrane staining dyes, such as MembraneBlue Dual (Dutch Ophthalmic Research Center (DORC), Zuidland, the Netherlands) or ILM-BLUE dye (DORC) were adopted to stain either the ERM or the ILM before peeling with forceps. At the end of the surgery, the eye was filled with a tamponade agent (air, fluid, or sulfur hexafluoride), and all the patients received a subconjunctival injection of an anti-inflammatory agent and antibiotics, abiding by the standard protocols.

## 4. Statistical Analysis

Continuous variables were reported as mean (±SD) and categorical features were reported as count (frequency). The normal distribution of continuous variables was verified with the Kolmogorov Smirnov test. Analysis of variance (ANOVA) and post-hoc analysis with Bonferroni and Games Howell tests were performed according to the results of Levene’s test homogeneity of variance to assess changes in both CRT and BCVA throughout the follow-up, based on EIFL presence. Paired samples *t*-tests were used to show correlations between mean changes of MS, fixation stability at 2° and 4° (FS 2° and FS 4°), and mfERG P1 wave in the central 4 rings (αP1-1; αP1-2; αP1-3; αP1-4) at baseline and at 12 months after surgery. Multi-variate linear regression analysis was used to determine significant independent predictors of visual acuity among demographics, cataract surgery and EIFL presence. Statistical analysis was performed using IBM SPSS software (IBM Corp., Armonk, NY, USA, version 28.0). Results were considered statistically significant when **p** value was <0.05.

## 5. Results

27 eyes of 27 patients, 12 females (44.4%) and 15 males (55.6%) affected by idiopathic ERM were retrospectively evaluated for 12 months after undergoing surgery. 

The mean age was 69.04 ± 7.44 years and mean disease duration was 18.85 ± 8.54 months.

Patients were categorized into 2 groups: group 1 (12 patients) was diagnosed with EIFL extending continuously from the INL and IPL across the fovea, while group 2 (15 patients) was characterized by EIFL absence on OCT scans. ERM and ILM were successfully removed in all 27 patients, as confirmed by OCT images. 

At baseline, 15 were phakic (55.6%) and 12 patients were pseudo-phakic (44.4%), whereas no one was aphakic. Therefore, the 12 pseudo-phakic eyes underwent vitrectomy only, while the 15 phakic (7 in group 1 and 8 in group 2) received a combined pars plana vitrectomy along with cataract surgery. 

**Figure 1:**
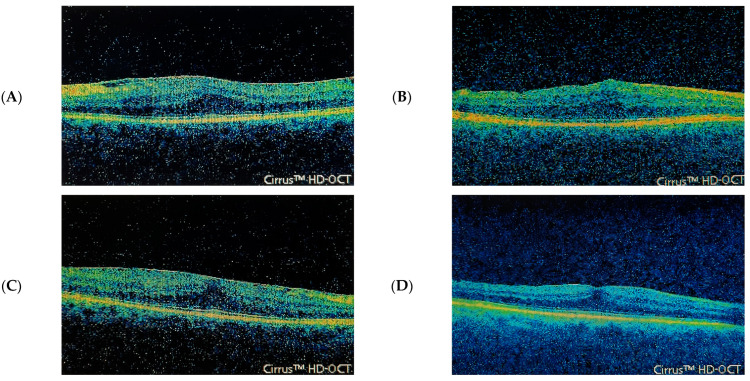
A. SD OCT images of two representative patients, the first of which (pictures (**A**) and (**B**), before and after surgery, respectively) was diagnosed with epiretinal membrane and ectopic inner foveal layers, as represented by the continuous hypo/hyperreflective band extending from the inner nuclear layer (INL) and inner plexiform layer (IPL) across the fovea. The second patient (pictures (**C**) and (**D**), before and after surgery, respectively) was instead diagnosed with epiretinal membrane but no ectopic inner foveal layers at both baseline and at 1-month postoperative follow-up, featuring only foveal pit absence with a substantially well-defined retinal architecture.

Two representative cases of patients from each group 1 and 2 are shown in Figure 1.

Table 2, Table 3 and Table 4 report a complete list of our findings, depending on EIFL presence.

In both groups 1 and 2, a significant (*p* < 0.05) improvement of BCVA was observed at 4 months (Group 1 = Mean Difference (MD): 0.14; Standard Error (SE): 0.04; Group 2 = MD: 0.31; SE: 0.07) and 12 months (Group 1 = MD: 0.13; SE: 0.05; Group 2 = MD 0.31; SE: 0.08) after treatment. Statistically significant differences were found between the two groups at all follow-up timepoints (*p* < 0.05). In a multivariate linear regression analysis, controlling for other possible variables affecting BCVA improvement, the presence of EIFL was the only independent predictive factor for lower visual acuity (*p* < 0.001).

**Figure 2:**
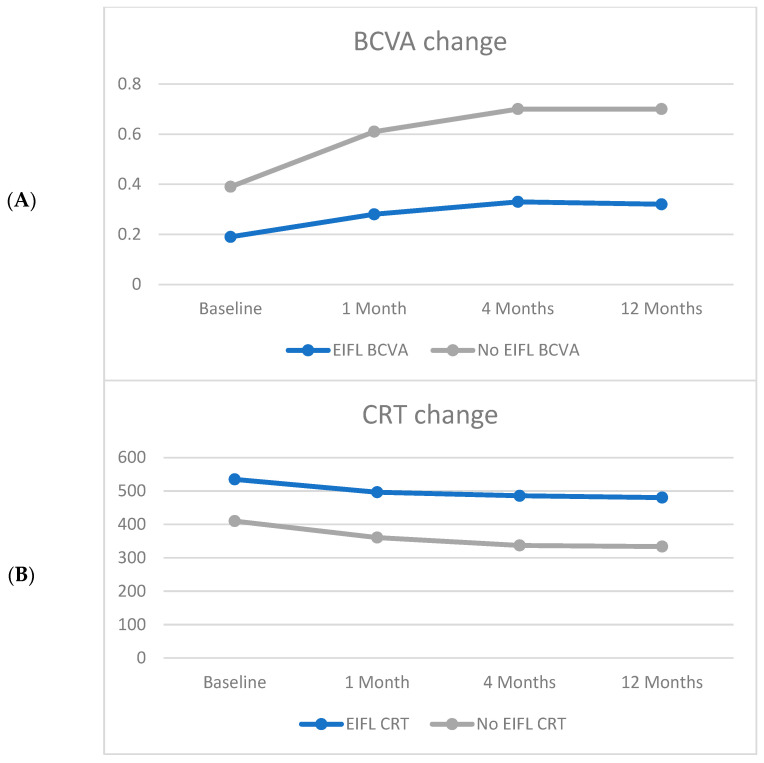
Line chart analysis of BCVA and CRT variation over time in both EIFL and No EIFL groups (Group 1 and 2, respectively). (**A**) A significant BCVA improvement was observed in both subgroups, with group 2 improving more than twofold compared to EIFL patients both at 4 and 12 months. (**B**) With regards to CRT, EIFL absence demonstrated a pivotal role in structural recovery, as thickness markedly shrunk throughout the follow-up in group 2, whereas no significant changes were found in group 1.

With regards to CRT, group 1 displayed no significant changes (*p* > 0.05) at any postoperative follow-up, whereas significant thickness decrease was found at the 4-month (MD: −73.13; SE: 23.56) and at the 12-month (MD: −76.20; SE: 23.56) follow-ups in group 2 (Figure 2).

MS showed no significant changes in both groups after surgery (*p* = 0.14 and *p* = 0.18 for group 1 and 2, respectively). 

FS at 2° and 4° showed no significant differences pre- and post-operatively in group 1 (*p* = 0.21 and *p* = 0.31, respectively), whereas group 2 displayed positive improvement in either FS 2° (+8.91 ± 13.97, *p* = 0.04) and FS 4° (+4.33 ± 3.84, *p* = 0.02) after 12 months from surgery. 

As for the P1 wave, Group 1 demonstrated no significant improvements within the 4 central rings after surgery. On the contrary, mfERG demonstrated a completely opposite trend in group 2, with highly significant (*p* < 0.001) improvements occurring in each ring (αP1-2: 27.97 ± 27.62; αP1-3: 12.51 ± 1 7.36; αP1-4: 10.49 ± 17.19) except for the most central (αP1-1), which did not reach statistical significance. 

## 6. Discussion

Vitreoretinal surgery through pars plana vitrectomy is the current mainstay for ERM treatment [16]. However, the optimal timing of surgery and the postoperative visual prognosis can be particularly challenging to predict [16]. 

Even though the current indication for surgical treatment focuses on the onset of metamorphopsia and on visual acuity worsening, increasing interest is growing in other biomarkers to better forecast ERM post-operative results. Among the several biomarkers advocated in the recent literature, great attention was given to the alterations of the inner retinal layers [7,8].

Therefore, in the present study, we evaluated the postoperative outcomes of our cohort of 27 patients diagnosed with idiopathic ERM by stratifying them on the basis of presence or absence of EIFL. 

According to previous reports, our results confirm a solid correlation between EIFL absence in preoperative OCT scans and better results in terms of BCVA improvement and CRT decrease during the 12-month post operative follow up [11,12].

In particular, BCVA displayed a significant recovery in both groups, with group 2 improving more than twofold compared to EIFL patients both at 4 and 12 months.

With regards to CRT, EIFL absence demonstrated a pivotal role in structural recovery, as thickness markedly shrunk throughout the follow-up in group 2, while no significant changes were found in group 1. 

The presence of EIFL may also impair the visual acuity by damaging the Muller cells-mediated normal intraretinal architecture, consequently compromising the bipolar cells’ functionality and the physiological neural transmission [17]. Indeed, all the patients underwent microperimetry evaluation, which provides information on macular function by assessing foveal fixation and macular sensitivity. It was previously demonstrated that long-term follow-up with microperimetry after idiopathic ERM surgery could be a valuable option to assess retinal sensitivity changes even when BCVA and CMT remain stable [18]. On the contrary, our results demonstrated no significant MS changes in both groups 1 and 2 before and after surgery, which is also in contrast to the results described by Mavi Yildiz et al., who reported a statistically significant improvement in case of EIFL absence [12].

We also conducted a study of FS, which is, to the best of our knowledge, the first carried out in consideration of the EIFL. FS at 2° and 4° showed no significant differences before and after surgery in group 1, whereas the absence of EIFL correlated with an evident improvement in both FS at 2° and at 4° after 12 months from surgery. 

Moreover, as far as we are concerned, this study also analyzed for the first time the impact of the presence of preoperative EIFL on the mfERG responses, which typically consist in a biphasic wave with an initial negative, followed by a positive and another negative deflection [19]. These three peaks are termed N1, P1 and N2, respectively, and the positive deflection seems to be generated from the inner retinal layer [19]. Thus, analyzing the P1 wave, the group 1 showed no significant improvement within the four central rings after surgery. On the contrary, mfERG demonstrated a completely opposite trend in group 2, with a highly significant raise in amplitude occurring in each ring except for the most central, which did not reach significance.

Interestingly, some reports showed a decrease of mfERG response amplitudes throughout the post-operative courses of idiopathic ERM surgeries performed with ILM peeling [13,20].

In particular, Terasaki et al. demonstrated an evident decrease of P1 amplitude at 12 months after surgery compared to baseline [21]. To explain these results, the possibility of an induced damage to Muller and, consequently, to bipolar cells caused by the ILM peeling has been discussed [21]. On the contrary, Moschos et al. reported a significant improvement in postoperative response densities in all cases analyzed [22]. In addition, the authors pointed out that retinal responses can greatly differ among patients with the same BCVA: the reconstitution of the intraretinal neural network seems to be independent from the photoreceptors’ integrity and the mfERG responses appeared to improve throughout time, even though BCVA remains relatively stable [22].

Similarly, Koutsandrea et al. described improved mfERG outcomes at 12 months after ERM surgery, also finding a significant correlation with the improved BCVA, whereas no significant BCVA changes were found at both 3 and 6 months [23]. Additionally, the authors demonstrated a significant correlation between mfERG responses reduction and indocyanine green (ICG) concentrations, whose toxicity may be a parameter to consider when evaluating functional macular results [23]. Overall, considering the notable discrepancy of these contrasting findings and taking into account the clear split between the two sharp opposite trends of mfERG change over time within the two groups explored in our report, it could be of interest to understand whether EIFL presence might have acted as a confounding factor among those cohorts of patients. 

We are aware that our study had several limitations, including the retrospective nature of the analysis. Retrospective data collections are unavoidably more flawed than prospective studies. First, retrospective analyses are more prone to selection biases and, as a consequence, the enrolled cohort and its partition might not be representative of the actual population, both in demographics (e.g., age and gender) and in classification (e.g., ERM or EIFL stages). These factors, thereby, might negatively affect the potential application of the current study to different demographic and clinical settings. In addition, as with other retrospective analyses, our report may also be flawed by clinical variable collection inconsistency (for example, BCVA). Additional limitations would take also into account the number of patients lost to follow-up, the subjective interpretation of several data and the manual assessment of the SD-OCT features (e.g., CRT), as well as the lack of quantification and evaluation of visual symptoms, such as metamorphopsia and contrast sensitivity, both at baseline and throughout the entire follow-up.

## 7. Conclusions

According to the data obtained in our cohort, a multimodal approach would enable highlighting the negative prognostic impact of the preoperative presence of EIFL on ERM surgery outcomes.

Therefore, the preferable timing of ERM surgery would be in the early phases of the disease, which are substantially characterized by EIFL absence, in order to obtain better functional and morphological outcomes.

Indeed, considering the results observed, our overall results seemed to converge in suggesting that the preoperative preservation of intraretinal architecture may be a prognostic factor of utmost importance for both anatomic and functional results.

Considering all the aforementioned limitations of our study, we would encourage further prospective, longitudinal analyses with larger number of patients to better understand in further detail the actual effects of EIFL presence on ERM surgery in order to additionally optimize the surgical timing and the overall postoperative outcomes.

## Figures and Tables

**Table 1:** Demographic and clinical ophthalmic data of the patients enrolled in the study.

Characteristics			EIFL	No EIFL
Age—Mean (SD)		69.04 (7.44) years		
Gender	Male Female	15 (55.6%) 12 (44.4%)		
N. patients			12 (44.4%)	15 (55.6%)
Disease duration—Mean (SD)		18.85 (8.54) months		
Lens status	Phakic Pseudophakic	15 (55.6%) 12 (44.4%)		

EIFL: ectopic inner foveal layers; N.: number of; SD: standard deviation.

**Table 2:** BCVA and CRT variation over the follow-up, stratified upon the presence of EIFL.

		BCVA	*p*	CRT	*p*
Group 1	B.	0.19		534.67	
	1 MO	+0.09 (SE 0.04)	0.27	−38.50 (SE 29.26)	0.56
	4 MO	+0.14 (SE 0.04)	0.01	−49.08 (SE 27.03)	0.29
	12 MO	+0.13 (SE 0.04)	0.04	−54.42 (SE 28.41)	0.25
Group 2	B.	0.39			410.00
	1 MO	+0.22 (SE 0.06)	0.06	−49.47 (SE 24.61)	0.20
	4 MO	+0.31 (SE 0.08)	0.00	−73.13 (SE 24.68)	0.03
	12 MO	+0.31 (SE 0.09)	0.00	−76.20 (SE 24.11)	0.02

B.: baseline; *p*: *p*-value.

**Table 3:** MS, FS 2° and FS 4° variation over the follow-up, stratified upon the presence of EIFL.

		MS *	*p*	FS 2° *	*p*	FS 4° *	*p*
Group 1	B.	9.96		58.50.		83.17	
	12 MO	+0.49 (1.07)	0.14	+11.17 (15.97)	0.21	+4.83	0.31
Group 2	B.	13.06		68.00		91.50	
	12 MO	+1.22 (3.11)	0.18	+8.91 (13.97)	0.04	+4.33 (3.84)	0.02

In brackets, standard deviation is reported (SD) *.

**Table 4:** MfERG αP1-1, αP1-2, αP1-3 and αP1-4 waves’ amplitude variation over the follow-up, stratified upon the presence of EIFL.

		αP1-1	*p*	αP1-2	*p*	αP1-3	*p*	αP1-4	*p*
Group 1	B.	/		22.55		18.51		14.48	
	12 MO	/		+27.99	0.08	+12.51	0.11	+10.49	0.16
Group 2	B.	/		44.15		33.15		28.79	
	12 MO	/		+26.36	0.00	+21.48	0.00	+19.58	0.00

## Data Availability

The datasets used and/or analyzed during the current study are available from the corresponding author on reasonable request.
